# Draft Genome Sequence of Antarctic Methanogen Enriched from Dry Valley Permafrost

**DOI:** 10.1128/genomeA.01362-16

**Published:** 2016-12-08

**Authors:** Joy Buongiorno, Jordan T. Bird, Kirill Krivushin, Victoria Oshurkova, Victoria Shcherbakova, Elizaveta M. Rivkina, Karen G. Lloyd, Tatiana A. Vishnivetskaya

**Affiliations:** a Department of Microbiology, University of Tennessee, Knoxville, Tennessee, USA; b Institute of Physicochemical and Biological Problems in Soil Science, Russian Academy of Sciences, Pushchino, Russia; c Skryabin Institute of Biochemistry and Physiology of Microorganisms, Russian Academy of Sciences, Pushchino, Russia

## Abstract

A genomic reconstruction belonging to the genus *Methanosarcina* was assembled from metagenomic data from a methane-producing enrichment of Antarctic permafrost. This is the first methanogen genome reported from permafrost of the Dry Valleys and can help shed light on future climate-affected methane dynamics.

## GENOME ANNOUNCEMENT

Permafrost currently contributes nearly 25% of all naturally sourced methane (1), a value that is predicted to rise significantly in coming decades (2). However, methane accumulation in permafrost environments is complex and geographically variable, making the trajectory of climate-affected methane dynamics hard to predict. Late Pleistocene permafrost from the Miers Valley (McMurdo Dry Valleys) contained methane in shallow horizons, where isotopic signatures suggested biogenic methane sources (3). In order to determine the biogenicity and timing of methane accumulation, incubation experiments were conducted. Here, we announce a nearly complete genome binned from those methane-producing enrichments.

Anaerobic incubations of permafrost consisted of phosphate-buffered basal medium (4) and gas mixture of H_2_/CO_2_ (80/20) at 20°C. Methane production was first observed after one year of incubation and is ongoing today (11 years later). After seven years, samples were collected for metagenome sequencing. The total community genomic DNA from the enrichment was extracted using the PowerSoil DNA isolation kit (Mo Bio Laboratories, Inc., Carlsbad, CA, USA), and the DNA library was prepared using the TruSeq DNA sample prep kit version 2 without whole-genome amplification. The Illumina HiSeq 2000 platform was used to acquire paired-end 2 × 100-bp metagenomic reads. Adaptors and low-quality reads were trimmed with the Trimmomatic software (5). VizBin (6) was used to bin together contigs of similar coverage and *k*-mer frequency. Metagenomic binning resulted in recovery of a nearly complete methanogenic genome, determined to be 99.84% complete using the *Euryarchaeota*-specific marker set of housekeeping genes (7), with low contamination (1.41%) and 0% strain heterogeneity.

The genomic reconstruction contained 342 contigs over 1,000 bp in length, with an average coverage of 570× and 38% GC content. RNAmmer (8) identified the 16S rRNA sequence, which BLASTn analysis shows to have 97% nucleotide sequence identity and 100% coverage to *Methanosarcina lacustris*, a psychrotolerant methanogen isolated from a fen in Moscow (9). Close relatives are *M. subterranea* strain HC-2 and *M. soligelidi* strain DSM 26065, isolated from a deep-subsurface diatomaceous shale formation and Siberian permafrost-affected soil, respectively (10, 11).

Annotation of protein-coding sequences was conducted with Prokka (12). The genome contained 3,593 coding regions, 53 tRNAs, 11 predicted CRISPR regions, and several cytochromes. The entire operon encoding methyl coenzyme M reductase (Mcr) and genes for hydrogenotrophic methanogenesis (*fmd*, *ftr*, *mch*, *mtd*, *mer*, *mtrABCDEFGH*, and *hdrABCDE*) were present. Acetoclastic genes encoding carbon monoxide dehydrogenase, acetate kinase, acetyl-coenzyme A synthetase, phosphate acetyltransferase, and the acetyl-CoA decarbonylase/synthase complex provide evidence that this organism is capable of acetoclastic methanogenesis. Methanol metabolism genes encoding the three subunits of methanol—corrinoid protein comethyltransferase—show potential for growth with methanol. The genome contains monomethylamine methyltransferase and dimethylamine corrinoid protein genes, suggesting growth with methylamines. An incomplete formate dehydrogenase operon suggests that growth with formate is not likely.

The genome contains evidence for *de novo* unsaturated diether lipid construction through a functional mevalonate pathway, a signature of adaptation to permanently cold environments (13). DNA double-strand break repair Rad50 ATPase and several heat-shock proteins were detected, indicating that several defense strategies against environmental stress are available to this methanogen.

### Accession number(s).

This whole-genome shotgun project has been deposited at DDBJ/ENA/GenBank under the accession number MCHG00000000 for the entire metagenome and MDTP00000000 for the reconstructed genome. The versions described in this paper are versions MCHG01000000 and MDTP02000000, respectively.
